# Eating habits and nutritional knowledge among amateur ultrarunners

**DOI:** 10.3389/fnut.2023.1137412

**Published:** 2023-07-10

**Authors:** Aureliusz Kosendiak, Magdalena Król, Marta Ligocka, Marta Kepinska

**Affiliations:** ^1^Department of Physical Education and Sport, Wroclaw Medical University, Wrocław, Poland; ^2^Department of Pharmaceutical Biochemistry, Division of Biomedical and Environmental Analyses, Faculty of Pharmacy, Wroclaw Medical University, Wrocław, Poland; ^3^Students Scientific Association, Division of Biomedical and Environmental Analyses, Faculty of Pharmacy, Wroclaw Medical University, Wrocław, Poland

**Keywords:** amateur ultrarunners, food choices, KomPAN questionnaire, physical activity, nutrition for amateur athletes

## Abstract

**Introduction:**

Many studies concerning the diet of physically active people refer to individuals who run; however, the importance of nutrition in professional and amateur sports plays a different role. This study aimed to evaluate the nutritional behavior and knowledge of amateur ultrarunners. This study involved a group of 308 respondents (89 women and 219 men) aged 18 -65. It investigated the influence of the level of knowledge about nutrition, gender, education, and smoking on dietary food habits and eating frequency.

**Methods:**

The KomPAN questionnaire was used to determine the dietary habits, diet quality, lifestyle, and nutrition knowledge of ultramarathon runners. The nutrition knowledge influenced the eating habits and frequency of specific meals expressed as the Healthy Diet Index-10 (HDI-10) and Unhealthy Diet Index-14 (UDI-14).

**Results:**

In women with sufficient knowledge about nutrition, lower HDI-10 scores were observed compared to those with a good level of knowledge, while men did not show a similar relationship. However, the effect of smoking on the frequency of food intake in men was noted. Interestingly, male smokers had a lower UDI-14 score than non-smokers. Depending on the level of knowledge, female and male ultrarunners more often or less frequently used selected food products. In turn, no effect of education on the frequency of consumption of specific foods was observed.

**Discussion:**

Such different results are most likely caused by the specificity of the study group, which consisted of amateur runners. Additionally, the study looked at general eating habits, not those employed when preparing for marathons. In the future, more respondents should be surveyed, also taking into account nutrition during training.

## 1. Introduction

Ultramarathon runners, ultramarathoners, or ultrarunners participate in runs and races longer than 42,195 meters. They can be amateurs, recreational joggers, or professional athletes. Participation in such competitions is often motivated by the desire for personal achievements, general health, and psychological reasons (1). The age of participants varies greatly, but peak performances are often achieved at 35 or older (2). Additionally, men take part in such races more often than women, although the number of female participants has been increasing over the last years, reaching about 20% (3). Competitors derive satisfaction from covering such long distances. For example, an increase in their self-esteem has been noticed (1). However, ultramarathons can also cause stress and extreme strain on the body (4). Therefore, they require proper preparation and diet. Healthy nutrition has been shown to impact the results in ultra-endurance competitions (5, 6). During such races, there is a significant energy deficit, which is why a properly selected diet is so important. The source of energy supplied depends on the intensity of training. During less intense exercise, energy comes from fats (7), while during events, between them, and during very intense exercise, mainly from carbohydrates (6). Carbohydrates are obtained primarily from meals with a high glycemic index because they are an excellent source for synthesizing glycogen stored in muscles (8). This has been confirmed by studies, which showed a positive correlation between high carbohydrate consumption and the results in endurance sports (9).

Ultramarathon runners can suffer from many health problems. They often complain of gastrointestinal disorders, digestive problems (10), nausea (11), and bleeding (12, 13). They limit the performance of ultramarathon runners and are also often the reason for withdrawing from races (10, 14). There are also difficulties with maintaining homeostasis of the energy balance. Due to the high energy demand, there is a risk of an imbalance between its supply and actual energy expenditure. This may result in exercise-related hyponatremia or caloric deficit (2). Studies have shown that you should eat your favorite foods during competitions as they reduce the risk of digestive problems (15). Therefore, runners should consciously choose food products and know the main sources of energy in ultramarathons (16). However, it is not possible to fully meet the energy needs during the race, so participants must face weight loss during the run and an increased caloric deficit (17). This is also accompanied by dehydration (18). For this reason, they should drink plenty of fluids, but excessive drinking is not advisable (19). It can cause hyponatremia associated with excessive blood dilution or physical exertion (20).

Food choices are influenced by social, economic, and cultural factors (21). Gender has been found to impact eating behavior significantly. Women pay attention to the sensory attractiveness of food to a greater extent than men (22). Taking into account only the influence of gender, women more often believe that healthy eating is important. For men, the health aspect of food choices is less critical (23). This has an impact on the food choices made. Researchers have noticed this in women with higher education. At the same educational level, 85% of women chose healthy foods compared to 61% of men. Women eat more fruit, fiber, and less fat compared to men. It is also affected by upbringing. Additionally, it has been shown that women are more willing to introduce changes in their diet; it is particularly evident among people with low socioeconomic status (24).

Another aspect that significantly affects diet quality is the education level. Prior research supports that people with lower education show less interest in health (24) and have worse eating habits than those with higher education, who make better choices (25). Therefore, in better-educated people, the risk of being overweight and obese is lower in all ethnic groups (26).

Cigarette smoking also has an impact on diet. Active and passive smokers have shown higher consumption of processed foods and other unhealthy foods. Many studies indicate that the diet of smokers is poorer in valuable ingredients and of lower quality compared to non-smokers (27, 28). Yet another factor influencing eating habits is the socioeconomic situation. This has been confirmed by a study conducted on a group of students, which showed that students make food choices based on their financial situation. Individuals financially supported by or living with their parents eat healthier than their self-supporting peers. Other studies also suggest that socioeconomic status (lower education, limited income, unemployment) is associated with increased food insecurity, i.e., insufficient quality and quantity of nutritionally adequate food (29). They choose fast-food products because of their convenience and relatively low price (22). Additionally, individuals who live with roommates or family frequently have a better diet than people who live alone (30). Food security is defined as “the situation when all people, at all times, have physical, social and economic access to sufficient, safe and nutritious food that meets their dietary needs and food preference for an active and healthy life” (31). However, food security issues were not directly addressed in this study.

The level of nutritional knowledge also influences the choice of consumed products. It was found that the greater the nutrition knowledge, the better the eating habits (32, 33). The level of physical activity can affect the quality of the food eaten. Physical activity is associated with reduced unhealthy food intake, such as candy and fatty foods (34). However, amateur athletes are a particularly interesting group who do not always pay attention to choosing the right diet (35). This is because of their approach to sports, which they treat as a hobby rather than a profession. However, it should be noted that participation in amateur competitions is often associated with a healthy lifestyle, which positively affects physical and mental health (36). This study aimed to evaluate the nutritional behavior of amateur ultrarunners, both men and women. We examined the relationship between the level of knowledge about nutrition and the choice of specific products, both healthy and unhealthy. Because the human diet depends not only on the level of nutritional knowledge, we also assessed the impact of factors such as gender, smoking, and education on the food choices of ultramarathoners.

## 2. Materials and methods

### 2.1. Characteristics of the studied group

We conducted a cross-sectional study. The study covered 308 adult amateur runners, including 219 men and 89 women. Individuals who take up ultrarunning amateurishly or recreationally are often not affiliated with any sports clubs. They work in other fields and run in their free time as a hobby, including ultra distances. In our research, the distribution by gender is uneven, but other studies of ultrarunners also show a higher percentage of male engagement (37). Pregnant women were not included in the study. The characteristics of the research group are presented in Table 1.

**Table 1:** The descriptive information for study characteristics and selected characteristics by sex.

Parameter	Men *n* = 219	Women *n* = 89	*p*
Age [years] 1st quartile; Me; 3rd quartile	37;**43**; 48	35;**39**; 44	**0.001**
BMI [kg/m^2^] 1st quartile; Me; 3rd quartile	22.72;**24.49**; 25.66	20.31;**21.46**; 23.31	**<0.000**
HDI-10 index 1st quartile; Me; 3rd quartile	17.60;**25.00**; 33.10	20.90;**29.60**; 35.90	**0.005**
UDI-14 index 1st quartile; Me; 3rd quartile	8.00;**13.07**; 18.57	5.79;**8.93**; 14.14	**<0.000**
Domicile I – a city with over 500,000 inhabitants II – a city with 500,000–100,000 inhabitants III – a city with 100,000–50,000 inhabitants IV – a city with 50,000–20,000 inhabitants V – a city with 20,000–5,000 inhabitants VI – village	I – 60 (27.40%) II – 51 (23.29%) III – 23 (10.50%) IV – 25 (11.41%) V – 22 (10.05%) VI – 38 (17.35%)	I – 32 (35.95%) II – 13 (14.61%) III – 8 (8.99%) IV – 13 (14.61%) V – 10 (11.23%) VI – 13 (14.61%)	0.430
Subjective assessment of health I – very bad/bad II – neutrally III – well IV – very well	I – 2 (0.91%) II – 8 (3.65%) III – 95 (43.38%) IV – 114 (52.06%)	I – 1 (1.12%) II – 0 (0.00%) III – 49 (55.06%) IV – 39 (43.82%)	0.056
Cigarette smoking	Yes: 204 (94.01%) No: 13 (5.99%)	Yes: 81 (92.05%) No: 7 (7.95%)	0.662
Education degree I – primary education II – vocational education III – secondary education IV – higher education	I – 3 (1.38%) II – 3 (1.38%) III – 48 (22.01%) IV – 164 (75.23%)	I – 0 (0.00%) II – 1 (1.15%) III – 13 (14.94%) IV – 73 (83.91%)	0.413
Type of preferred training I – individual II – with a partner III – group	I – 170 (77.62%) II – 30 (13.70%) III – 19 (8.68%)	I – 64 (71.91%) II – 17 (19.10%) III – 8 (8.99%)	0.475
Number of competitions per year I – 0 II – 1 III – 2–5 IV – 6–10 V – 11–15 VI – 16–20 VII – 21–30	I – 10 (4.57%) II – 1 (0.46%) III – 97 (44.29%) IV – 78 (35.62%) V – 23 (10.50%) VI – 5 (2.28%) VII – 5 (2.28%)	I – 5 (5.62%) II – 1 (1.12%) III – 28 (31.46%) IV – 36 (40.45%) V – 10 (11.24%) VI – 4 (4.49%) VII – 5 (5.62%)	0.340
Average number of running kilometers per week 1st quartile; Me; 3rd quartile	50;**60**; 80	45;**50**; 70	**0.001**
The longest distance [km] 1st quartile; Me; 3rd quartile	65;**100**; 130	55;**75**; 110	0.001

Me – median; the longest distance – the greatest number of kilometers that the respondent managed to run at one time. Bold values represent median and statistical significance (*p* < 0.05).

### 2.2. Procedures

The research was conducted in a group of amateur ultrarunners from the beginning of January 2021 to the end of March 2021. This period included the nationwide lockdown in Poland, where, most of the time, all social meeting points were fully or partially excluded from use. It also covered the closure of sports clubs and gyms and limited access to outdoor activities. Certainly, during the lockdown, the characteristics of the group in terms of training would have looked different (limitations, bans, a general trend of reduced levels of physical activity) (38). Therefore runners were asked to evaluate the entire period of experience with ultrarunning.

KomPAN^®^ Dietary Habits and Nutrition Beliefs Questionnaire and eating habits questionnaires were used for the study. Additional questions covered ‘metrics’ and participants’ training characteristics, such as training duration, number of kilometers run per week, training time, etc. All questions were included in the Supplementary Questionnaire S1. Participation in the research was voluntary, anonymous, and approved by the Bioethics Committee of Wroclaw Medical University (No. KB-601/2021). Subjects gave informed consent to participate in the study included in the online questionnaire. These questionnaires are research tools often used in publications, and for this study, they were converted into online versions and distributed using Google Docs. Before using the questionnaire, the Google Docs were tested many times to ensure the feasibility of administering the questionnaire on every basic device and web browser. The lack of direct face-to-face interaction may have positively impacted the respondents, thanks to which they had a greater sense of anonymity and thus felt more at ease and did not censor their responses. The research was conducted asynchronously: the study participants had a lot of time to give answers, and they could do it at any time without time pressure. The questionnaire was available for several weeks to members of the focus groups.

The following criteria were adopted for the amateur ultrarunners: participation in ultra-competitions, taking up running for non-profit purposes, for health and personal satisfaction, overcoming one’s own weaknesses; training without the intervention of a professional coach; not being affiliated with a sports club; association to an amateur ultrarunning group on social media; having a paid job and treating running as a hobby.

**Figure 1:**
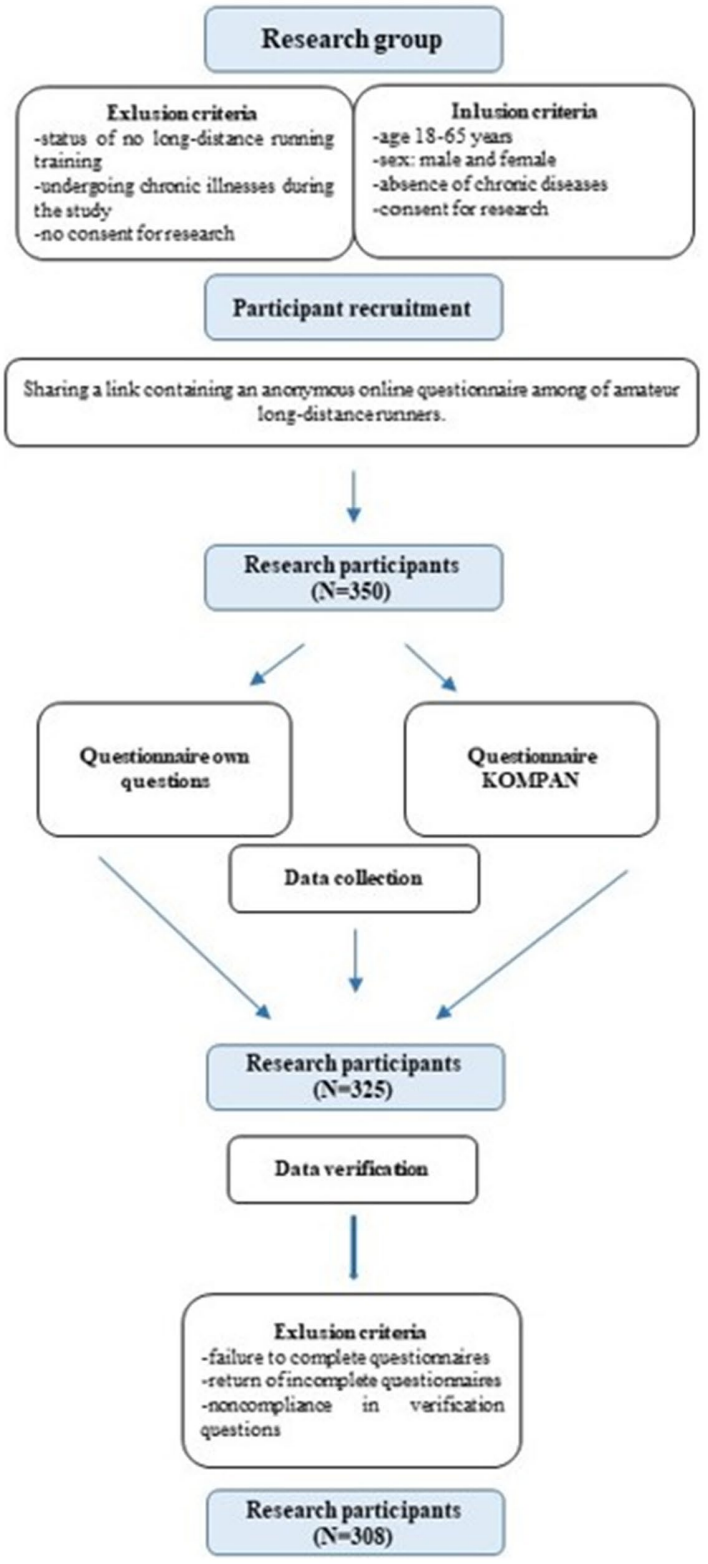
Selection process of the study.

A total of 450 links with the online questionnaire were sent out. In order to confirm the identity of the research participants, the links with the questionnaire were sent to groups of amateur ultrarunners, where individuals’ names are associated through social media. In order to obtain valuable data, the moderator and distributor of the questionnaires was a person known in the community with an ultrarunning background. In order to collect data from amateur ultrarunners, several questions were used to verify, among others, the number of completed ultra runs and runs over 42 km. This eliminated the possibility of sending the questionnaire to random people. In phase 1, which lasted a month, a total of 350 questionnaires were collected. Participants who provided unreliable answers or did not answer the questions comprehensively were removed. In phase 2, which lasted 2 months, 325 participants qualified after the first verification process. In phase 3, additional exclusion criteria (failure to complete questionnaires, return of incomplete questionnaires, non-compliance in verification questions, chronic diseases) were introduced (19, 23). After the entire verification process, 308 respondents were accepted. The process of preparation and selection of study participants is shown in the diagram (Figure 1). The respondents were asked to fill in online questionnaires using Google Docs. It is a platform for online file transfer and collaborative editing. The respondents received a link redirecting them to a relevant online questionnaire in Google Docs. Part of the questions was self-reported, containing basic information about the study participants: marital status, age, gender, height and weight, smoking, socioeconomic status, education level, and place of residence according to city size. The body mass index (BMI) was also used in the study. Measurements were not taken using the bioimpedance method due to the limitations of COVID-19, and body weight and height were self-reported. The self-reported survey also included questions about preferred forms of training, volume, frequency, and duration. In addition, questions covered training experience, the number of ultra runs, the average number of km run per week, and the longest distance run.

### 2.3. Dietary habits, diet quality, and nutrition knowledge

The research employed the KomPAN questionnaire developed by the Committee of Human Nutrition Science, Polish Academy of Sciences (39). The KomPAN questionnaire is a reliable, consistent, and authoritative tool for studying dietary beliefs and habits (40, 41). This has been confirmed by many papers in international journals by Polish and foreign scholars. The questionnaire was used to determine the dietary habits, diet quality, lifestyle, and nutrition knowledge of ultramarathon runners. Each respondent, on their own, filled in the questionnaire in the form of an online survey consisting of 111 questions divided into four main topics: dietary habits (11 questions), frequency of food consumption (33 questions), nutrition beliefs (25 questions), lifestyle and personal data. Then, following the KomPAN analysis instructions, four parameters were estimated: “Healthy Diet Index,” “Unhealthy Diet Index,” physical activity, and nutrition knowledge. Food frequency consumption was put into six categories [from “never” – (1) to “few times a day” – (6)]. The frequency of food consumption was also converted into “times per day” [from “never” – (0) to “few times a day – (2)]. Diet scores were calculated based on daily food consumption. The Healthy Diet Index-10 (HDI-10) was calculated as the sum of the frequency of eating healthy food groups included in 10 questions (23, 25, 31–33, 37, 38, 40, 42–43; the total score range: 0–20 points; Supplementary Table S1). The Unhealthy Diet Index-14 (UDI-14) was calculated as the sum of the frequency of eating unhealthy food groups included in 14 questions (22, 24, 26–29, 34–36, 44, 46, 51–52, 54; the total score range: 0–28 points; Supplementary Table S2). Then these were summed up and classified according to the scale: HDI-10: low = 0–6.66; moderate = 6.67–13.33; high = 13.34–20; UDI-14: low = 0–9.33; moderate = 9.34–18.66; high = 18.67–28. Nutrition knowledge was estimated on the basis of 25 questions from Part C of the questionnaire. For each correct answer, the participant received 1 point, and at the end, the result was summed up and classified: insufficient = 0–8; sufficient = 9–16; good = 17–25.

### 2.4. Statistical analyses of data

The analyses were carried out using the STATISTICA 13.3 (Statsoft Polska, Sp. z o.o.) package under Wroclaw Medical University’s license. The normality of the distribution of variables was checked using the Shapiro–Wilk test, and the homogeneity of variance was examined using Levene’s test. Due to the lack of normality of all distributions, it was decided to use non-parametric tests. The Mann–Whitney *U* test was employed to determine statistically significant differences between the two groups, and the Kruskal-Wallis *H* test and the *post hoc* Dunn’s multiple comparisons test were utilized for more than two groups. Spearman’s rank order test was used for correlation analysis. GraphPad Prism 9 (GraphPad Software) was used to visualize the data. Statistical significance was assumed for *p* < 0.05.

## 3. Results

### 3.1. The relationship between the Healthy Diet Index-10 (HDI-10), the Unhealthy Diet Index-14 (UDI-14), and nutrition knowledge

In the group of women, nutrition knowledge influenced the HDI-10 scores but not the UDI-14 scores (Table 2). Women with sufficient knowledge about nutrition achieved lower HDI-10 scores compared to women whose level of knowledge was assessed as good (*p* = 0.032). Women with insufficient knowledge also obtained lower scores than those with good knowledge, but these differences were not statistically significant. In the case of men, no similar relationships were observed.

**Table 2:** Modeling results of HDI-10 and UDI-14 by the level of nutritional knowledge.

The level of nutritional knowledge	Women			
	HDI-10 index 1st quartile; Me; 3rd quartile	p (Kruskal-Wallis *H* test)	UDI-14 index 1st quartile; Me; 3rd quartile	p (Kruskal-Wallis *H* test)
Good	29.60;**35.50**; 41.40	**0.016**	5.36;**7.04**; 10.79	0.177
Sufficient	19.70;**28.40**; 34.90*		6.36;**12.07**; 15.36	
Insufficient	16.60;**22.30**; 34.20		5.00;**8.68**; 10.57	
The level of nutritional knowledge	Men			
	HDI-10 index 1st quartile; Me; 3rd quartile	p (Kruskal-Wallis H test)	UDI-14 index 1st quartile; Me; 3rd quartile	p (Kruskal-Wallis H test)
Good	22.10;**27.80**; 35.30	0.167	8.36;**12.36**; 17.93	0.224
Sufficient	17.40;**24.90**; 32.80		7.86;**13.11**; 18.29	
Insufficient	15.55;**20.95**; 30.10		10.54;**16.21**; 22.79	

* Significant difference compare to the good level of knowledge (*p* = 0.032, Dunn’s test). Bold values represent median and statistical significance (*p* < 0.05).

### 3.2. The relationship between the Healthy Diet Index-10 (HDI-10), the Unhealthy Diet Index-14 (UDI-14), and smoking

Smoking cigarettes did not affect the HDI-10 and UDI-14 scores in the group of women (Table 3). However, in the group of men, the UDI-14 scores were significantly lower among smoking respondents compared to non-smokers (*p* = 0.032).

**Table 3:** Modeling results of HDI-10 and UDI-14 by the cigarette smoking.

Cigarette smoking	Women			
	HDI-10 index 1st quartile; Me; 3rd quartile	p (Mann–Whitney *U* test)	UDI-14 index 1st quartile; Me; 3rd quartile	p (Mann–Whitney *U* test)
Yes	20.90;**29.60**; 35.90	0.711	5.36;**7.04**; 10.79	0.694
No	16.90;**35.30**; 42.80		3.79;**7.79**; 14.36	
Cigarette smoking	Men			
	HDI-10 index 1st quartile; Me; 3rd quartile	p (Mann–Whitney *U* test)	UDI-14 index 1st quartile; Me; 3rd quartile	p (Mann–Whitney *U* test)
Yes	17.80;**25.30**; 33.20	**0.128**	7.96;**12.89**; 18.21*	**0.032**
No	14.80;**19.60**; 26.40		13.64;**19.00**; 22.79	

* Significant difference compare to the non-smoking group (*p* = 0.032, Mann–Whitney *U* test). Bold values represent median and statistical significance (*p* < 0.05).

### 3.3. The relationship between the Healthy Diet Index-10 (HDI-10), the Unhealthy Diet Index-14 (UDI-14), and education

In both groups (women and men), no significant relationship was found between the level of education and the scores of the studied indices (HDI-10 and UDI-14). However, among men, the difference in the HDI-10 scores between secondary and higher education was on the verge of statistical significance (*p* = 0.066). The results are presented in Table 4.

**Table 4:** Modeling results of HDI-10 and UDI-14 by the education degree.

Education degree	Women			
	HDI-10 index 1st quartile; Me; 3rd quartile	p (Mann–Whitney *U* test)	UDI-14 index 1st quartile; Me; 3rd quartile	p (Mann–Whitney *U* test)
Primary education	x	0.190	x	0.227
Vocational education	x		x	
Secondary education	17.00;**23.70**; 35.60		8.29;**13.14**; 15.14	
Higher education	21.90;**30.40**; 36.70		5.64;**8.79**; 14.14	
Education degree	Men			
	HDI-10 index 1st quartile; Me; 3rd quartile	p (Kruskal-Wallis *H* test)	UDI-14 index 1st quartile; Me; 3rd quartile	p (Kruskal-Wallis *H* test)
Primary education	19.20;**24.20**; 29.20	0.066	7.57;**13.79**; 22.50	0.594
Vocational education	14.80;**23.20**; 25.30		4.79;**7.00**; 15.07	
Secondary education	15.55;**21.90**; 28.90		7.86;**12.68**; 18.61	
Higher education	18.90;**26.35**; 34.05		8.46;**13.21**; 18.64	

Bold values represent median and statistical significance (*p* < 0.05).

### 3.4. The relationship between nutrition knowledge and the frequency of healthy food consumption

**Figure 2:**
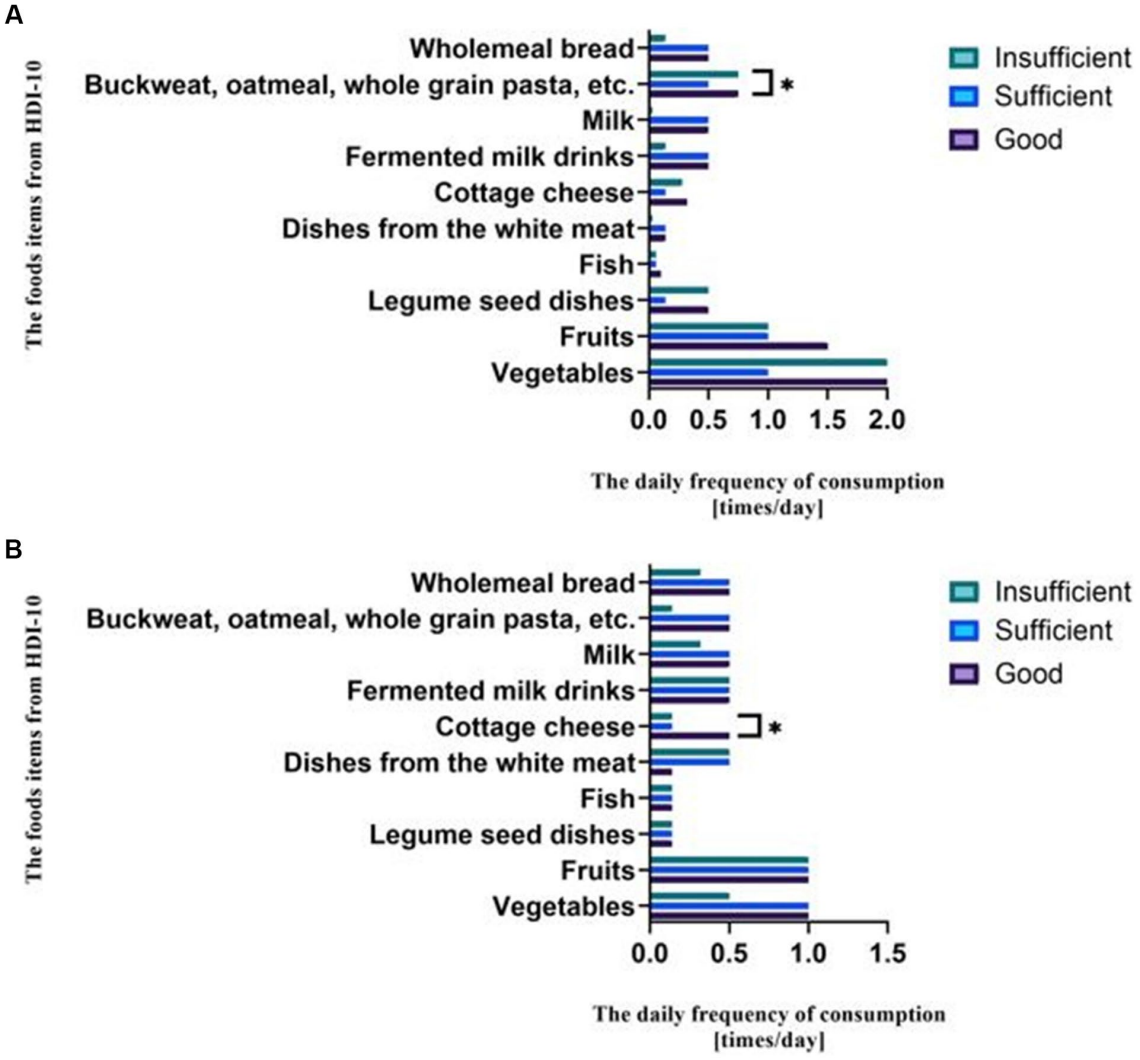
**(A)** The association between the frequency of consuming food items from the HDI-10 and the level of nutritional knowledge among women. * Significant difference between the groups (*p* = 0.011, Kruskal Wallis *H* test). **(B)** The association between the frequency of consuming food items from the HDI-10 and the level of nutritional knowledge among men. * Significant difference between the groups (*p* = 0.038, Kruskal Wallis *H* test).

Women with sufficient and good nutrition knowledge consumed statistically greater amounts of products such as buckwheat, whole-grain pasta, oatmeal, and other coarse grains. Women with insufficient knowledge consumed statistically less of them (Figure 2A). Men with good nutrition knowledge consumed statistically more cottage cheese. Men with sufficient and insufficient nutrition knowledge consumed this product in smaller amounts (Figure 2B).

### 3.5. The relationship between nutrition knowledge and the frequency of unhealthy food consumption

**Figure 3:**
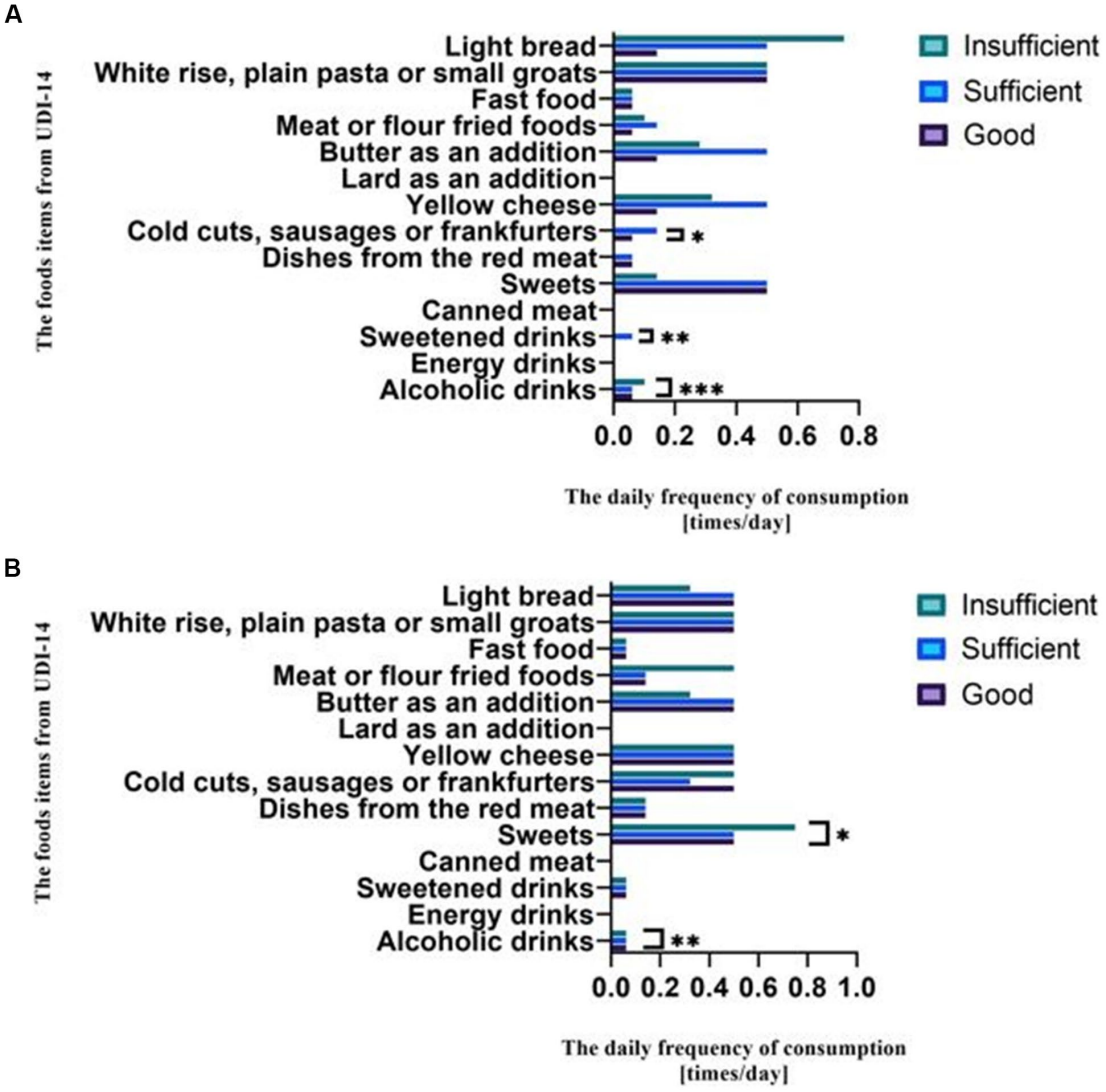
**(A)** The association between the frequency of consuming food items from the UDI-14 and the level of nutritional knowledge among women. * Significant difference between the groups (*p* = 0.014, Kruskal Wallis *H* test). ** Significant difference between the groups (*p* = 0.039, Kruskal Wallis *H* test). *** Significant difference between the groups (*p* = 0.042, Kruskal Wallis *H* test). **(B)** The association between the frequency of consuming food items from the UDI-14 and the level of nutritional knowledge among men. * Significant difference between the groups (*p* = 0.025, Kruskal Wallis *H* test); ** Significant difference between the groups (*p* = 0.026/Kruskal Wallis *H* test).

Women with sufficient nutritional knowledge consumed statistically more products, such as cold cuts, sausages, or frankfurters, than women with good and insufficient dietary knowledge. Consumption of products from the group of sweetened drinks was found to be statistically significant only in women with sufficient nutritional knowledge. Women with insufficient nutritional knowledge used alcoholic drinks more often than women with good and sufficient knowledge (Figure 3A). Men with insufficient knowledge consumed statistically more sweets compared to men with good and sufficient knowledge. A similar relationship occurred with alcoholic drinks (Figure 3B).

### 3.6. Correlation between selected features and the HDI-10 or UDI-14 among men and women

In the group of women, no correlation was found between the selected features and the HDI-10 and UDI-14. In men, a negative correlation (*r* = −0.162, *p* = 0.017) was found between the running training period and the frequency of reaching for healthy products (HDI-10). There was also a positive correlation (*r* = 0.143, *p* = 0.035) between the number of runs over 43 kilometers per year and unhealthy eating habits. The results are presented in Table 5.

**Table 5:** The correlations between selected characteristics and the HDI-10 index or the UDI-14 index.

Correlations	Women			
	HDI-10		UDI-14	
	*R*	*p*	*R*	*p*
Age	0.125	0.242	−0.060	0.574
Cigarette smoking	0.055	0.606	−0.050	0.643
Running training period (in years)	0.107	0.320	−0.098	0.363
Number of runs over 43 km per year	0.049	0.648	0.022	0.835
Number of competitions per year	0.157	0.142	−0.126	0.239
Average number of running kilometers per week	0.038	0.726	0.047	0.946
Number of trainings per week	0.087	0.421	−0.031	0.772
The longest distance	−0.080	0.456	0.127	0.237
Correlations	Men			
	HDI-10		UDI-14	
	*R*	*p*	*R*	*p*
Age	−0.059	0.382	−0.016	0.813
Cigarette smoking	0.063	0.350	0.049	0.472
Running training period (in years)	−0.162	**0.017**	−0.025	0.718
Number of runs over 43 km per year	−0.102	0.133	0.143	**0.035**
Number of competitions per year	−0.087	0.198	0.062	0.365
Average number of running kilometers per week	0.047	0.489	0.036	0.596
Number of trainings per week	0.044	0.521	−0.011	0.874
The longest distance	0.023	0.732	−0.003	0.967

*R* – Spearman’s rank correlation coefficient; *p* – significance level. Statistically significant correlations are in bold.

## 4. Discussion

In today’s world, participation in ultrarunning is becoming more and more popular, also among amateurs (42). However, the mere desire to do sports does not give people sufficient knowledge about proper and rational nutrition (43). Other factors influence the choice of the right diet. Therefore, this study aimed to examine the impact of nutritional knowledge, smoking, and education on the frequency of consuming healthy and unhealthy products. Due to awareness of the influence of gender on food choices, the respondents were divided into two groups: men and women.

The nutritional knowledge of the respondents was measured using the KomPAN questionnaire and categorized as good, sufficient, or insufficient. The analysis of the results showed a relationship between nutritional knowledge and the frequency of meals consumed. In the case of women, lower knowledge was associated with a lower index corresponding to a healthy diet. In turn, in the case of men, lower knowledge about nutrition was associated with a higher index corresponding to an unhealthy diet. In both cases, the same trend is noticeable. This is consistent with the results of other studies, according to which people who perform better on a test related to nutrition knowledge are more likely to eat healthy food (44, 45). On the other hand, some unhealthy products (sausages, frankfurters, etc.) were more often chosen by women with sufficient nutritional knowledge compared to women with insufficient knowledge. This may be because the respondents have never been under the care of a dietitian, and their dietary choices may be based on their own beliefs or experience. Research shows that even some athletes in sports clubs have never had dietary consultations, which may significantly affect the frequency of choosing specific food products (46).

Individuals practicing sports are often involuntarily associated with healthy habits, i.e., they consume little alcohol or do not smoke cigarettes (47). Meanwhile, studies have repeatedly shown that anti-healthy behaviors also often appear in people practicing sports (48, 49). This study’s results indicate that smoking men are less likely to reach for unhealthy products than non-smokers. However, it should be remembered that the vast majority of respondents were smokers, which may significantly affect the results of this study. However, an interesting observation arises here that amateur ultrarunners are still mostly susceptible to unhealthy addictions, despite practicing sports.

The assessment of the relationship between food choices and education was also the subject of this research. The level of education can affect diet quality, food choices, and the diet itself (50). Among the surveyed group of amateur ultramarathoners, 71.1% were men. Both among women and men, most of the respondents had higher education. Despite numerous studies showing that women care more about their diet and food choices (23, 51), no statistically significant differences were found between the level of education and the frequency of healthy eating habits in women training ultrarunning in this study. It was similar in the case of men, where there was no correlation between the level of education and the HDI-10 and UDI-14 scores (Table 4).

In addition to the quantity and quality of nutritional products consumed, frequency of consumption is also important. This study shows that women with sufficient nutritional knowledge consumed statistically more products such as cold cuts, sausages, or frankfurters than women with good and insufficient nutritional knowledge. Consumption of sweetened beverages was statistically significant only in women with sufficient nutritional knowledge. Women with insufficient nutritional knowledge used alcoholic beverages more often than women with good and sufficient knowledge (Figure 3A). Men with insufficient knowledge consumed statistically more sweets than men with good and sufficient knowledge. A similar relationship occurred with alcoholic beverages (Figure 3B).

On the one hand, the results indicate that individuals with less knowledge about nutrition may more often choose unhealthy products, which is confirmed in the literature (52). However, on the other hand, it should be noted that the vast majority of respondents obtained only sufficient results regarding nutrition knowledge. This indicates that despite the respondents doing sports and trying to lead a healthy lifestyle, they do not necessarily have adequate nutrition knowledge. In the case of amateurs, nutrition and nutritional knowledge do not necessarily have to be related and correlated with the achievement of specific sports results but rather constitute a mechanism leading to the achievement of generally understood good health, which is confirmed by other studies (53). For example, it would seem that prolonged training and frequent heavy competition should promote unhealthy eating habits in men (Table 5). However, it should be remembered that the research was carried out on a group of amateurs who, despite doing sports, do not necessarily eat healthy food. Amateurs undertaking ultramarathon training and competing for recreation have increased energy expenditure (54). To balance it, they must ensure an adequate supply of energy. It is unimportant for them to achieve good results in the competition, so they allow themselves to deviate from healthy food. They often exert themselves to a higher level of energy expenditure, which is then covered by unhealthy snacks and other unhealthy products (55, 56). For professional runners, proper nutrition and diet can affect athletic performance, which is the main goal, in contrast to amateur runners (57). Although ultrarunning training does not promote unhealthy dietary choices, it should be noted that unhealthy dietary choices do occur among amateur runners, as confirmed by the results of our study (55, 58).

However, this research has some limitations, including unequal gender distribution. As mentioned before, men dominate among ultrarunners (3, 37), as confirmed by this study. The study group itself is also not too large, but this may be due to the period in which the study was conducted (the beginning of the COVID-19 lockdown in Poland). People were forced to put their usual hobbies on hold, so they talked less about them on social media (59). In the future, similar studies should take into account a longer period given to complete the questionnaire but also attempt to reach ultrarunners not only online but also through sports clubs. It would be interesting to check the eating habits of runners during training, and not just between them. The study also did not consider SES (60), which could have significantly impacted the respondents’ food choices. Similarly, the study did not take into account issues related to food security, which could also translate into the respondents’ use of a particular diet. SES and food security should also be included in future research to understand these relationships better. This research is a preliminary study; however, despite some limitations, it shows in a fairly extensive way the dependence of dietary choices of amateur ultrarunners on various factors. Expanding this topic could draw runners’ attention to their nutrition.

## 5. Conclusion

Maintaining a healthy diet is especially important for people practicing sports; therefore, nutritional knowledge is at a higher level in people who are more active and have higher education compared to people who are less physically active and have lower education.

Both in the case of women and men, the level of nutrition knowledge was associated with changes in the HDI-10 and UDI-14 scores. Women with sufficient knowledge about nutrition obtained statistically lower HDI-10 scores compared to women with good nutrition knowledge. However, no similar relationship was found in men. There was also no significant effect of education on food choices and the frequency of consumption of specific products. Interestingly, it was noticed that male smokers had lower UDI-14 scores.

Such results may be due to the specifics of the study group, which consisted of amateur ultrarunners, which means that they are not necessarily well-educated in nutrition and positive health behavior. It was similar in the case of the selection of food products (from the healthy and unhealthy categories), where nutrition knowledge did not always correspond with the healthiest choices. For example, regardless of the level of knowledge, men consumed UDI-14 products such as white rice, plain pasta, small groats, yellow cheese, or sweetened drinks with the same frequency. In turn, regardless of the level of knowledge, women consumed HDI-10 products such as fermented milk drinks, fish, legume seed dishes, or fruit with the same frequency. This may be because, for amateurs practicing sports, diet is not always put in the first place, as the physical activity and the fun that comes from it are much more important.

## Data availability statement

The raw data supporting the conclusions of this article will be made available by the authors, without undue reservation.

## Ethics statement

The studies involving human participants were reviewed and approved by the Bioethics Committee at the Wroclaw Medical University (no. KB-601/2021). The patients/participants provided their written informed consent to participate in this study.

## Author contributions

AK and MKe contributed to conception and design of the study. AK organized the database. MKr performed the statistical analysis. AK, MKr, ML, and MKe wrote sections of the manuscript. All authors contributed to manuscript revision, read, and approved the submitted version.

## Funding

The presented research results, implemented under the topic according to the records in the SIMPLE system with the number SUBZ.Z604.23.006 were financed from the subvention awarded by the Minister of Science and Higher Education, Poland.

## Conflict of interest

The authors declare that the research was conducted in the absence of any commercial or financial relationships that could be construed as a potential conflict of interest.

## Publisher’s note

All claims expressed in this article are solely those of the authors and do not necessarily represent those of their affiliated organizations, or those of the publisher, the editors and the reviewers. Any product that may be evaluated in this article, or claim that may be made by its manufacturer, is not guaranteed or endorsed by the publisher.
