# Supplementary material for: Eating habits and nutritional knowledge among amateur ultrarunners
Source: Front Nutr. 2023 Jul 10;10:1137412. doi: 10.3389/fnut.2023.1137412 (PMC10365969; doi:10.3389/fnut.2023.1137412)
Supplement: Supplementary file 1 [file Data_Sheet_1.pdf]

Supplementary Material

Eating habits and nutritional knowledge among amateur ultra-runners

Aureliusz Kosendiak, Magdalena Król, Marta Ligocka, Marta Kepinska*

*** Correspondence:** Marta Kepinska: marta.kepinska@umw.edu.pl

1 Supplementary Questionnaire

Questionnaire S1. Questionnaire for examining views and eating habits for people aged 16 to 65

1. Respondent code

2. Interviewer code

3. Resort code

4. A day of research

5. A month of research

6. A year of research

General questions: (Own questions)**:**

a) Age (years)
b) gender
c) place of residence
d) Body weight [kg]
e) Body high[cm]
f) How many times per week you are running
g) How do you subjectively assess your health status?
h) What is your average number of running miles per week?

i) What is your average average training time per week? (in hours)
j) What was the longest distance you ran? (in kilometers)
k) How many competitions do you compete in each year?
l) For what purpose do you compete in running competitions?
m) Do you participate in competitions/street races, etc.?
n) How long have you been training in running(in years)?

Part A. Eating habits

7. How many meals do you usually eat per day?
   (1) 1 meal
   (2) 2 meals
   (3) 3 meals
   (4) 4 meals
   (5) 5 meals or more

8. Do you eat meals at fixed times of the day?
   (1) No
   (2) Yes, but only some meals
   (3) Yes, all meals

9. How often do you eat food between meals?
   (1) Never
   (2) 1-3 times a month
   (3) Once a week
   (4) A few times a week
   (5) Once a day

10. What food do you usually eat between meals on weekdays?
   (1) Fruits
   (2) Vegetables
   (3) Unsweetened milk drinks and desserts, e.g. yoghurts, cottage cheese, milk
   (4) Sweetened milk drinks and desserts, e.g. cream cheese, sweetened milk drinks, flavoured milk
   (5) Sweet snacks, e.g. candies, cookies, cakes, chocolate bars, muesli bars, wafers
   (6) Salty snacks, e.g. crackers, sticks, chips, fries
   (7) Nuts, almonds, seeds, pits
   (8) Other products. Which ones? ………………………………………………………..

11. What kind of milk and milk drinks do you consume most often?
   (1) Standard fat (full fat)
   (2) With reduced fat content
   (3) Without fat

12. How do you usually eat prepared meat dishes?
    (1) Cooked
    (2) Stewed
    (3) Grilled
    (4) Baked
    (5) Fried
    (6) I don't eat meat

13. What kind of fat do you use most often for spreading bread?
    (1) I don't use any fat on bread
    (2) I use a variety of fats
    (3) Mayonnaise
    (4) Margarine
    (5) Butter
    (6) Mix butter with margarine
    (7) Lard

14. What kind of fat do you use most often for frying food?
    (1) I do not use any fat to fry food
    (2) I use a variety of fats
    (3) Vegetable oil (including olive oil)
    (4) Margarine
    (5) Butter
    (6) Lard

15. Do you sweeten hot drinks, e.g. tea, cocoa, coffee?
    (1) No
    (2) Yes, I sweeten with one teaspoon of sugar (or honey)
    (3) Yes, I sweeten with two or more teaspoons of sugar (or honey)
    (4) Yes, I use sweeteners (low energy sweeteners)

16. Do you add ready meals and sandwiches at the table?
    (1) No
    (2) Yes, but only sometimes
    (3) Yes, I add salt to most foods

17. What kind of water do you usually drink?
    (1) I don't drink water
    (2) I drink still water
    (3) I drink sparkling water
    (4) I drink flavoured water

18. What day of the week was it?
    (1) Monday

(2) Tuesday
(3) Wednesday
(4) Thursday
(5) Friday
(6) Saturday
(7) Sunday

19. How many meals did you eat on this day? ……………………………………………….

20. How many times did you eat vegetables or fruits on this day? …………………………...

21. Did you eat fast food on this day, e.g. fries, hamburgers, pizza, hot dogs, casseroles?
    (1) No
    (2) Yes. How many times? …………………………………………………………………

Part B. Frequency of food consumption

22. How often do you eat light bread, e.g. wheat, rye, mixed wheat-rye, toast bread, rolls, croissants?
    (1) Never
    (2) 1-3 times a month
    (3) Once a week
    (4) Several times a week
    (5) Once a day
    (6) Several times a day

23. How often do you eat wholemeal bread?
    (1) Never
    (2) 1-3 times a month
    (3) Once a week
    (4) Several times a week
    (5) Once a day
    (6) Several times a day

24. How often do you eat white rice, plain pasta or small groats, e.g. semolina, couscous?
    (1) Never
    (2) 1-3 times a month
    (3) Once a week
    (4) Several times a week
    (5) Once a day
    (6) Several times a day

25. How often do you eat buckwheat, oatmeal, whole wheat pasta or other coarse grains?
(1) Never
(2) 1-3 times a month
(3) Once a week
(4) Several times a week
(5) Once a day
(6) Several times a day

26. How often do you eat fast food, e.g. french fries, hamburgers, pizza, hot dogs, casseroles?
(1) Never
(2) 1-3 times a month
(3) Once a week
(4) Several times a week
(5) Once a day
(6) Several times a day

27. How often do you eat fried foods (e.g. meat or flour)?
(1) Never
(2) 1-3 times a month
(3) Once a week
(4) Several times a week
(5) Once a day
(6) Several times a day

28. How often do you consume butter as an addition to bread or dishes, for frying, baking, etc.?
(1) Never
(2) 1-3 times a month
(3) Once a week
(4) Several times a week
(5) Once a day
(6) Several times a day

29. How often do you eat lard as an addition to bread or dishes, for frying, baking, etc.?
(1) Never
(2) 1-3 times a month
(3) Once a week
(4) Several times a week
(5) Once a day
(6) Several times a day

30. How often do you consume oils or margarines or mixtures of butter and margarine, as an addition to bread or dishes, for frying, baking, etc.?
(1) Never
(2) 1-3 times a month

(3) Once a week
(4) Several times a week
(5) Once a day
(6) Several times a day

31. How often do you consume milk (including flavoured milk, cocoa, milk coffee)?
(1) Never
(2) 1-3 times a month
(3) Once a week
(4) Several times a week
(5) Once a day
(6) Several times a day

32. How often do you consume fermented milk drinks, e.g. yoghurts, kefirs (natural or flavoured)?
(1) Never
(2) 1-3 times a month
(3) Once a week
(4) Several times a week
(5) Once a day
(6) Several times a day

33. How often do you eat cottage cheese (including homogenized cheese, curd desserts)?
(1) Never
(2) 1-3 times a month
(3) Once a week
(4) Several times a week
(5) Once a day
(6) Several times a day

34. How often do you eat cheese (including processed cheese, blue cheese)?
(1) Never
(2) 1-3 times a month
(3) Once a week
(4) Several times a week
(5) Once a day
(6) Several times a day

35. How often do you eat cold cuts, sausages or frankfurters?
(1) Never
(2) 1-3 times a month
(3) Once a week
(4) Several times a week
(5) Once a day
(6) Several times a day

36. How often do you eat foods from red meat, e.g. pork, beef, veal, mutton, lamb, venison?
(1) Never
(2) 1-3 times a month
(3) Once a week
(4) Several times a week
(5) Once a day
(6) Several times a day

37. How often do you eat foods from white meat, e.g. chicken, turkey, rabbit?
(1) Never
(2) 1-3 times a month
(3) Once a week
(4) Several times a week
(5) Once a day
(6) Several times a day

38. How often do you eat fish?
(1) Never
(2) 1-3 times a month
(3) Once a week
(4) Several times a week
(5) Once a day
(6) Several times a day

39. How often do you eat eggs?
(1) Never
(2) 1-3 times a month
(3) Once a week
(4) Several times a week
(5) Once a day
(6) Several times a day

40. How often do you eat legumes, e.g. beans, peas, soybeans, lentils?
(1) Never
(2) 1-3 times a month
(3) Once a week
(4) Several times a week
(5) Once a day
(6) Several times a day

41. How often do you eat potatoes (not including french fries and crisps)?
(1) Never
(2) 1-3 times a month
(3) Once a week

(4) Several times a week
(5) Once a day
(6) Several times a day

42. How often do you eat fruit?
(1) Never
(2) 1-3 times a month
(3) Once a week
(4) Several times a week
(5) Once a day
(6) Several times a day

43. How often do you eat vegetables?
(1) Never
(2) 1-3 times a month
(3) Once a week
(4) Several times a week
(5) Once a day
(6) Several times a day

44. How often do you eat sweets, e.g. candies, cookies, cakes, chocolate bars, muesli bars, other confectionery?
(1) Never
(2) 1-3 times a month
(3) Once a week
(4) Several times a week
(5) Once a day
(6) Several times a day

45. How often do you eat powdered soups or ready-made soups, e.g. from a can, jar, thickened (not including frozen soups)
(1) Never
(2) 1-3 times a month
(3) Once a week
(4) Several times a week
(5) Once a day
(6) Several times a day

46. How often do you eat canned meat?
(1) Never
(2) 1-3 times a month
(3) Once a week
(4) Several times a week
(5) Once a day

(6) Several times a day

47. How often do you eat canned vegetables, pickled or pickled vegetables?
(1) Never
(2) 1-3 times a month
(3) Once a week
(4) Several times a week
(5) Once a day
(6) Several times a day

48. How often do you drink fruit juices?
(1) Never
(2) 1-3 times a month
(3) Once a week
(4) Several times a week
(5) Once a day
(6) Several times a day

49. How often do you drink vegetable or fruit-vegetable juices?
(1) Never
(2) 1-3 times a month
(3) Once a week
(4) Several times a week
(5) Once a day
(6) Several times a day

50. How often do you drink sweetened hot drinks such as tea, coffee, herbal or fruit infusions?
(1) Never
(2) 1-3 times a month
(3) Once a week
(4) Several times a week
(5) Once a day
(6) Several times a day

51. How often do you drink sweetened carbonated or non-carbonated drinks such as Coca-Cola, Pepsi, Sprite, Fanta, orangeade, lemonade?
(1) Never
(2) 1-3 times a month
(3) Once a week
(4) Several times a week
(5) Once a day
(6) Several times a day

52. How often do you drink energy drinks, e.g. 2 KC, Black Horse, Red Bull, Burn, Shot or others?
(1) Never

(2) 1-3 times a month
(3) Once a week
(4) Several times a week
(5) Once a day
(6) Several times a day

53. How often do you drink water, e.g. mineral, table water?
(1) Never
(2) 1-3 times a month
(3) Once a week
(4) Several times a week
(5) Once a day
(6) Several times a day

54. How often do you drink alcoholic beverages?
(1) Never
(2) 1-3 times a month
(3) Once a week
(4) Several times a week
(5) Once a day
(6) Several times a day

Part C. Views on food and nutrition

Content of the statement	(1) True	(2) False	(3) Hard to say
58. Eating moldy bread can cause Salmonella infection.			
59. High salt intake protects against high blood pressure.			
60. Reducing fatty foods in your diet helps prevent cardiovascular disease.			
61. Frequent consumption of fatty sea fish accelerates the development of atherosclerosis.			
62. Eating grilled meat promotes cancer.			
63. A consequence of a vegetarian diet is an increased risk of anaemia.			
64. Bio-yoghurts contain beneficial gut bacteria.			
65. Oil and olive oil contain a lot of cholesterol.			
66. Wholemeal bread contains more fiber than white bread.			

67. Fruits and vegetables are a source of "empty calories".			
68. Butter and fortified margarines are high in vitamins A and D.			
69. Cheese is a better source of calcium than cottage cheese.			
70. Offal contains significant amounts of “bad” LDL cholesterol.			
71. Complex carbohydrates should be replaced with simple sugars in the diet.			
72. Protein should be the main source of energy in a proper diet.			
73. Insufficient intake of vitamin PP can cause dermatitis and diarrhoea.			
74. Staying in the sun promotes the production of vitamin D in the body.			
75. Phosphorus is a component of nervous tissue.			
76. The ratio of calcium to phosphorus in a proper diet should be 1:1.			
77. Eating fruits rich in vitamin C increases the absorption of iron.			
78. Starting to cook vegetables in cold water helps to preserve their nutritional value.			
79. Sweets and animal fats are distinguished by their high nutritional density.			

Part D. Lifestyle and personal data

80. Do you currently follow any diet?
    (1) No
    (2) Yes, on the order of a doctor for health reasons
    (3) Yes, I follow a diet of my own choice

81. Please indicate the type of diet: ………………………………………………………….

82. How long have you been following the diet? ……………………………………………

83. How often do you eat meals outside your home, e.g. in bars, restaurants, cafes, canteens?
    (1) Never
    (2) 1-3 times a month
    (3) Once a week
    (4) Several times a week
    (5) Once a day
    (6) Several times a day

84. What alcoholic beverage do you drink most often?
(1) Beer
(2) Wine
(3) Drinks
(4) Strong drinks

85. Do you currently smoke cigarettes, a pipe or other tobacco?
(1) No
(2) Yes

86. Have you smoked cigarettes, a pipe or other tobacco in the past?
(1) No
(2) Yes

87. How many hours a day do you spend on average sleeping on weekdays?
(1) 6 or less hours/day
(2) 7 or 8 hours/day
(3) 9 and more hours/day

88. How many hours a day do you spend on average sleeping on weekends?
(1) 6 or less hours/day
(2) 7 or 8 hours/day
(3) 9 and more hours/day

89. How many hours a day do you spend on average watching TV or in front of a computer (including work)?
(1) Less than 2 hours
(2) From 2 to almost 4 hours
(3) From 4 to almost 6 hours
(4) From 6 to almost 8 hours
(5) From 8 to almost 10 hours
(6) 10 hours and more

90. How do you rate your physical activity at work or at school?
(1) Small: more than 70% of the time seated
(2) Moderate: about 50% of the time sitting and about 50% of the time moving
(3) High: about 70% of the time in motion or strenuous physical work

91. How do you rate your physical activity in your free time?
(1) Small: mostly sitting, watching TV, reading newspapers, books, light housework, walking 1-2 hours a week
(2) Moderate: walking, cycling, gymnastics, gardening, or other light physical activity performed 2-3 hours a week

(3) High: cycling, running, gardening or gardening, and other recreational sporting activities involving physical exertion more than 3 hours per week

92. How do you rate your health compared to other people of the same age?
    (1) Worse than peers
    (2) Same as peers
    (3) Better than peers

93. How do you rate your nutritional knowledge?
    (1) Insufficient
    (2) Sufficient
    (3) Good
    (4) Very good

94. How would you rate your eating habits?
    (1) Very bad
    (2) Bad
    (3) Good
    (4) Very good

95. How do you rate your eating habits on weekdays compared to weekends?
    (1) It's basically no different
    (2) It differs slightly
    (3) It differs significantly

96. What is your body weight in kg? ……………………………………………………….............

97. What is your height in cm? …………………………………………………………………

98. What is your waist circumference in cm? …………………………………………………….

99. Sex:
    (1) Male
    (2) Woman

100. Date of birth: …………………………………………………………………………….

101. Month of birth: …………………………………………………………………………..

102. Year of birth: ……………………………………………………………………………

103. What is your permanent residence?
    (1) Village
    (2) City under 20,000 inhabitants
    (3) City from 20,000 up to 100 thousand inhabitants

(4) City over 100,000 inhabitants

104. How many people are in your household (including yourself)? …………………………

105. How many minors are there in your household? ………………………………………...

106. How do you assess your financial situation?
(1) Below average
(2) Average
(3) Above average

107. How do you assess the situation of your household?
(1) We live very poor – we do not even have enough for basic needs
(2) We live modestly – we have to manage very economically every day
(3) We live on average – it's enough for us every day, but we have to save for more serious purchases
(4) We live well – enough for us without much saving
(5) We live very well – we can afford some luxury

108. Do you work professionally?
(1) No, I am retired or on disability
(2) No, I am on parental leave, I am unemployed, I am a housewife
(3) Yes, but I work part-time
(4) Yes, I have a steady job
(5) No, I am studying or studying

109. What is your education?
(1) Primary education
(2) Vocational education
(3) Secondary education
(4) Higher education

110. What is your mother's (caregiver's) education?
(1) Primary education
(2) Vocational education
(3) Secondary education
(4) Higher education

111. What is your father's (guardian's) education?
(1) Primary education
(2) Vocational education
(3) Secondary education
(4) Higher education

2 Supplementary Tables

Table S1. KomPAN Questionnaire – food products from the HDI-10 index with a potentially beneficial effect on health.

Nr	Healthy Diet Index (HDI-10)
**1**	Wholemeal bread
**2**	Buckwheat, oatmeal, whole grain pasta or other coarse grains
**3**	Milk (including flavoured milk, cocoa, coffee pudding)
**4**	Fermented milk drinks, e.g. yoghurts, kefirs (natural or flavoured)
**5**	Cottage cheese (including homogenized cheese, cottage cheese desserts)
**6**	Dishes from the so-called white meat, e.g. chicken, turkey, rabbit
**7**	Fish
**8**	Legume seed dishes, e.g. beans, peas, soybeans, lentils
**9**	Fruits
**10**	Vegetables
HDI-10 = sum of the frequency of consumption of 10 food groups (times/day; range: 0-20)	

Table S2. KomPAN Questionnaire – food products from the UDI-14 index with a potentially harmful effect on health.

**Nr**	**Unhealthy Diet Index (UDI-14)**
**1**	Light bread, e.g. wheat, rye, mixed wheat-rye, toasted bread, rolls, croissants
**2**	White rice, plain pasta or small groats, e.g. semolina, couscous
**3**	Fast food such as chips, burgers, pizza, hot dogs, casseroles
**4**	Meat or flour fried foods
**5**	Butter as an addition to bread or dishes, for frying, baking, etc.
**6**	Lard as an addition to bread or dishes, for frying, baking, etc.
**7**	Yellow cheese (including processed cheese, blue cheese)
**8**	Cold cuts, sausages or frankfurters
**9**	Dishes from the so-called red meat, e.g. pork, beef, veal, lamb, lamb, game
**10**	Sweets, e.g. candies, cookies, cakes, chocolate bars, muesli bars, other confectionery
**11**	Canned meat
**12**	Sweetened carbonated or non-carbonated drinks such as Coca-Cola, Pepsi, Sprite, Fanta, orangeade, lemonade
**13**	Energy drinks, e.g. 2 KC, Black Horse, Red Bull, Burn, Shot or others
**14**	Alcoholic drinks
UDI-14 = sum of the frequency of consumption of 14 food groups (times/day; range: 0-28)	

Table S3. Recommended values of the frequency of consumption of food products from the KomPAN questionnaire.

The frequency of consumption	Daily frequency (times/day)
Never	0
1-3 times a month	0.06
Once a week	0.14
A few times a week	0.5
Once a day	1
Several times a day	2

Table S4. Recommended method of the HDI-10 index and the UDI-14 index interpretation for the KomPAN questionnaire.

The intensity of nutritional characteristics	Range (times/day)	
	Healthy Diet Index, HDI-10	Unhealthy Diet Index, UDI-14
**Low**	0-6.66	0-9.33
**Medium**	6.67-13.33	9,34-18.66
**High**	13.34-20	18.67-28
